# Retraction: Time series expression pattern of key genes reveals the molecular process of esophageal cancer

**DOI:** 10.1042/BSR20191985_RET

**Published:** 2026-09-17

**Authors:** Jiafu Wang, Xiang Xie, Yurong Sun

**Affiliations:** 1 Department of Cardiothoracic Surgery, Shanxian Central Hospital, Heze 274300, Shandong Province, China; 2 Department of Gastroenterology, Shanxian Central Hospital, Heze 274300, Shandong Province, China; 3 Department of Surgical Care, Shanxian Central Hospital, Heze 274300, Shandong Province, China

**Keywords:** esophageal cancer, gene interaction module, methylation, regulatory factors

This article is being retracted from *Bioscience Reports* at the request of the Editor-in-Chief and the Editorial Board. This follows an internal investigation that identified multiple similarities between the Figures of this article with those published in Liu et al. 2019 (DOI: 10.3748/wjg.v25.i48.6890). Specifically, these include:

- Figure 1A with Figure 1B from Liu et al. 2019
- Figure 1C with Figure 1A from Liu et al. 2019
- Figure 2B with Figure 2A from Liu et al. 2019
- Figure 3A with Figure 2B from Liu et al. 2019
- Figure 3B with Figure 2C from Liu et al. 2019

The authors have been contacted with regard to the concerns and retraction but have not responded. Given the extent of the issues raised, the Editorial Board stands by the decision to retract the article.

